# Impaired auditory discrimination and auditory-motor integration in hyperfunctional voice disorders

**DOI:** 10.1038/s41598-021-92250-8

**Published:** 2021-06-23

**Authors:** Defne Abur, Austeja Subaciute, Mara Kapsner-Smith, Roxanne K. Segina, Lauren F. Tracy, J. Pieter Noordzij, Cara E. Stepp

**Affiliations:** 1 Department of Speech, Language, and Hearing Sciences, Boston University, Boston, MA 02215 USA; 2 Department of Biomedical Engineering, Boston University, Boston, MA 02215 USA; 3 Department of Speech and Hearing Sciences, University of Washington, Seattle, WA 98195 USA; 4 Department of Otolaryngology - Head and Neck Surgery, Boston University School of Medicine, Boston, MA 02118 USA

**Keywords:** Auditory system, Motor control, Sensorimotor processing, Sensory processing

## Abstract

Hyperfunctional voice disorders (HVDs) are the most common class of voice disorders, consisting of diagnoses such as vocal fold nodules and muscle tension dysphonia. These speech production disorders result in effort, fatigue, pain, and even complete loss of voice. The mechanisms underlying HVDs are largely unknown. Here, the auditory-motor control of voice fundamental frequency (*f*_o_) was examined in 62 speakers with and 62 speakers without HVDs. Due to the high prevalence of HVDs in singers, and the known impacts of singing experience on auditory-motor function, groups were matched for singing experience. Speakers completed three tasks, yielding: (1) auditory discrimination of voice *f*_o_; (2) reflexive responses to sudden *f*_o_ shifts; and (3) adaptive responses to sustained *f*_o_ shifts. Compared to controls, and regardless of singing experience, individuals with HVDs showed: (1) worse auditory discrimination; (2) comparable reflexive responses; and (3) a greater frequency of atypical adaptive responses. Atypical adaptive responses were associated with poorer auditory discrimination, directly implicating auditory function in this motor disorder. These findings motivate a paradigm shift for understanding development and treatment of HVDs.

## Introduction

Approximately 30% of individuals will develop a voice disorder across their lifetime^1^, and hyperfunctional voice disorders (HVDs) are the most common clinical diagnosis^2^. HVDs are primarily characterized by increased laryngeal muscle tension^3,4^ and can present with or without fibrovascular lesions on the vocal folds^5^. These dysregulations of laryngeal muscle tension are accompanied by changes in vocal quality i.e., a strained and/or breathy voice^3,6^, fatigue^7^, pain^8^, and aphonia complete loss of voice^9^, which result in voice changes that interfere with communication and affect quality of life (e.g., job retention^1^).

The primary symptoms of HVDs are related to speech motor production, such that the amount of voice use is likely related to their development^3^. Some have also postulated that there are links between psychosocial behavior^10,11^ and autonomic dysfunction^12,13^ and HVDs. Overall, the mechanisms underlying HVDs are still largely unknown. This gap in understanding is an obstacle in determining clear diagnostic^14,15^, assessment^16,17^, and therapeutic protocols^18,19^, and often results in a diagnosis of exclusion (i.e., individuals receive a HVD diagnosis if there are no other clear structural or neurological reasons for the voice changes^20^). This has led to speculation that there may be subtypes of HVDs^14,21,22^. Although HVDs are characterized by impaired voice production, recently, early work has suggested that atypical auditory function may be a key determinant in their development in some individuals with HVDs^23–25^.

The generation of appropriate laryngeal muscle control patterns depends on the ability to detect somatosensory and auditory changes and generate corrective output; therefore, persistent improper laryngeal control in individuals with HVDs may result from atypical auditory-motor function. Typical auditory-motor control of voice relies on: (1) the detection of auditory errors (the mismatch between expected and actual feedback); (2) generation of a corrective motor plan by the auditory feedback system; and (3) updating of the feedforward system to incorporate the corrective plan into future utterances^26^. Experimentally, shifts (i.e., alterations) can be applied to auditory feedback of fundamental frequency (*f*_o_; the acoustic correlate of pitch) to create a mismatch between expected and actual feedback (i.e., an auditory ‘error’) in order to examine all three components of auditory-motor control in the voice domain. First, comparisons of shifted and non-shifted voice *f*_o_ can be used in listening tasks to find auditory discrimination thresholds^27,28^. Second, sudden, unexpected shifts in auditory *f*_o_ feedback yield ‘reflexive’ responses, which provide information about corrective vocal motor plans^24,29–31^. Third, sustained, predictable shifts in auditory *f*_o_ feedback yield ‘adaptive’ responses, which demonstrate whether the feedforward system has incorporated corrections and updated subsequent vocal motor plans (auditory-motor integration^25,32^).

In separate investigations, previous work has implicated potential disruptions to auditory-motor control of voice in HVDs at all three levels: detection of auditory errors, generation of corrective motor plans, and updating of the feedforward system. In a pure tone pitch discrimination task, 24 speakers with HVDs showed worse detection thresholds compared to 63 controls, which suggests deficits in auditory error detection in individuals with HVDs^23^. However, hearing status and singing experience were not reported. These are important considerations, with evidence supporting increased prevalence of hearing impairment in individuals with HVDs^33^ and benefits of musicality for pitch discrimination^34^. Another study reported greater *f*_o_ reflexive response magnitudes in 10 speakers with HVDs compared to 17 controls. However, the sample size was small and the study did not include singers, who have an increased risk for developing HVDs^1^ and have shown differences in auditory-motor control (including reduced adaptive responses and reduced reflexive responses compared to non-singers^32,35^). Finally, a preliminary study reported a larger proportion of atypical adaptive *f*_o_ responses in 9 individuals with HVDs compared to 9 control speakers^25^, although the small sample size limits the generalizability of these findings. In summary, previous work evaluated disparate aspects of speech motor control in modest cohorts of speakers with HVDs, resulting in a patchwork of information that suggests a potential auditory-motor basis for HVDs.

The current study goal was to comprehensively examine auditory-motor control of voice *f*_o_ in a large sample of individuals with HVDs and controls (matched for hearing status and singing experience). We investigated auditory discrimination, reflexive responses, and adaptive responses in 124 speakers (62 with HVDs). We hypothesized that, compared to controls and regardless of singing experience, individuals with HVDs would show: (1) worse auditory discrimination; (2) greater reflexive response magnitudes; and (3) greater occurrences of atypical adaptive responses.

## Results

### Auditory discrimination

**Figure 1:**
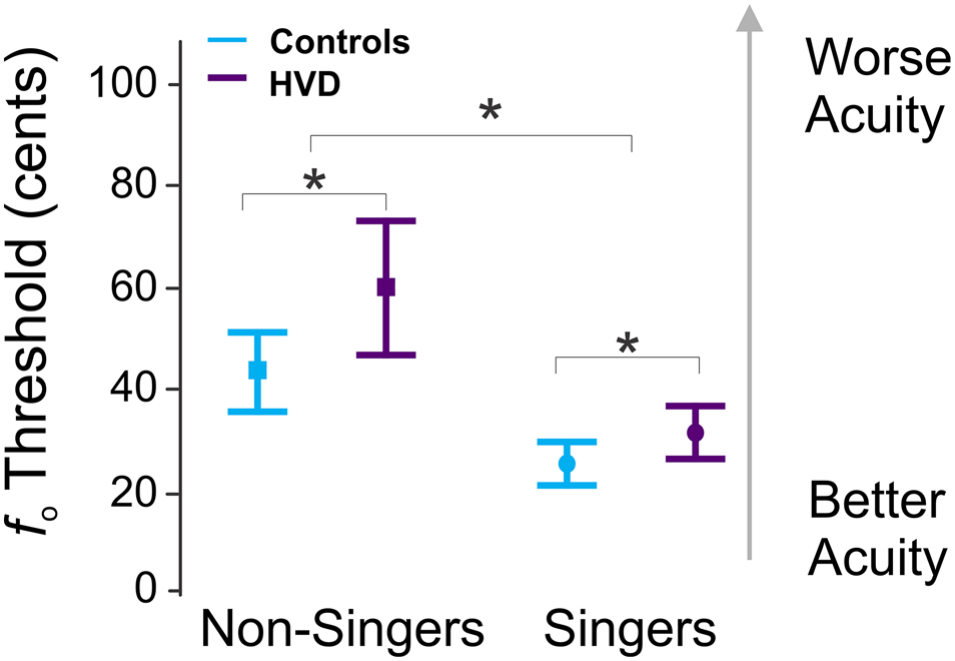
Auditory discrimination. The average auditory discrimination thresholds for changes in voice fundamental frequency (*f*_o_) in cents are shown for the control group non-singers (light blue square, left; N = 33), HVD group non-singers (dark purple square, left; N = 33), control group singers (light blue circle, right; N = 29) and HVD group singers (dark purple circle, right; N = 29). The error bars indicate 95% confidence intervals. *Indicates statistical significance at the *p* < .05 level.

A two-way analysis of variance (ANOVA) revealed a significant effect of group (HVD vs. controls; *df* = 1, F = 7.32, *p* = .008; small-to-medium effect size, η_p_^2^ = 0.06) and singing experience (singers vs. non-singers; *df* = 1, F = 29.43, *p* < .001; medium-to-large effect size, η_p_^2^ = 0.2) on auditory discrimination thresholds (Fig. 1). The interaction between group and singing experience was not statistically significant (*df* = 1, F = 1.40, *p* = .239). Post-hoc Tukey tests demonstrated that individuals with HVDs had statistically worse discrimination (M = 47 cents, SD = 32 cents) compared to the control group (M = 35 cents, SD = 20 cents), and that singers had better discrimination (M = 28 cents, SD = 13 cents) compared to non-singers (M = 52 cents, SD = 31 cents). Summary statistics for all experimental tasks are shown in Table 1.

**Table 1:** Statistical results for each experimental task.

**Auditory discrimination results: two-way ANOVA**				
Factor	*df*	F	*p*	η_p_^2^
Group (HVD or control)	1	7.32	.008	0.06
Singing experience	1	29.43	< .001	0.2
Group × singing experience	1	1.40	.239	

**Reflexive response results: three-way mixed effects ANOVA**				
Factor	*df*	F	*p*	η_p_^2^
Group (HVD or control)	1	0.39	.535	
Singing experience	1	0.00	.951	
Shift direction (up or down)	1	22.99	< .001	0.06
Group × singing experience	1	0.82	.367	
Group × shift direction	1	1.2	.276	
Singing experience × shift direction	1	6.81	.010	0.02
Group × singing experience × shift direction	1	0.04	.848	

**Adaptive response results: Chi-squared test for association**				
*df*	*X*^2^	*p*	Cramer’s V	
3	14.02	.003	0.33	

*HVD* hyperfunctional voice disorder.

### Reflexive responses

**Figure 2:**
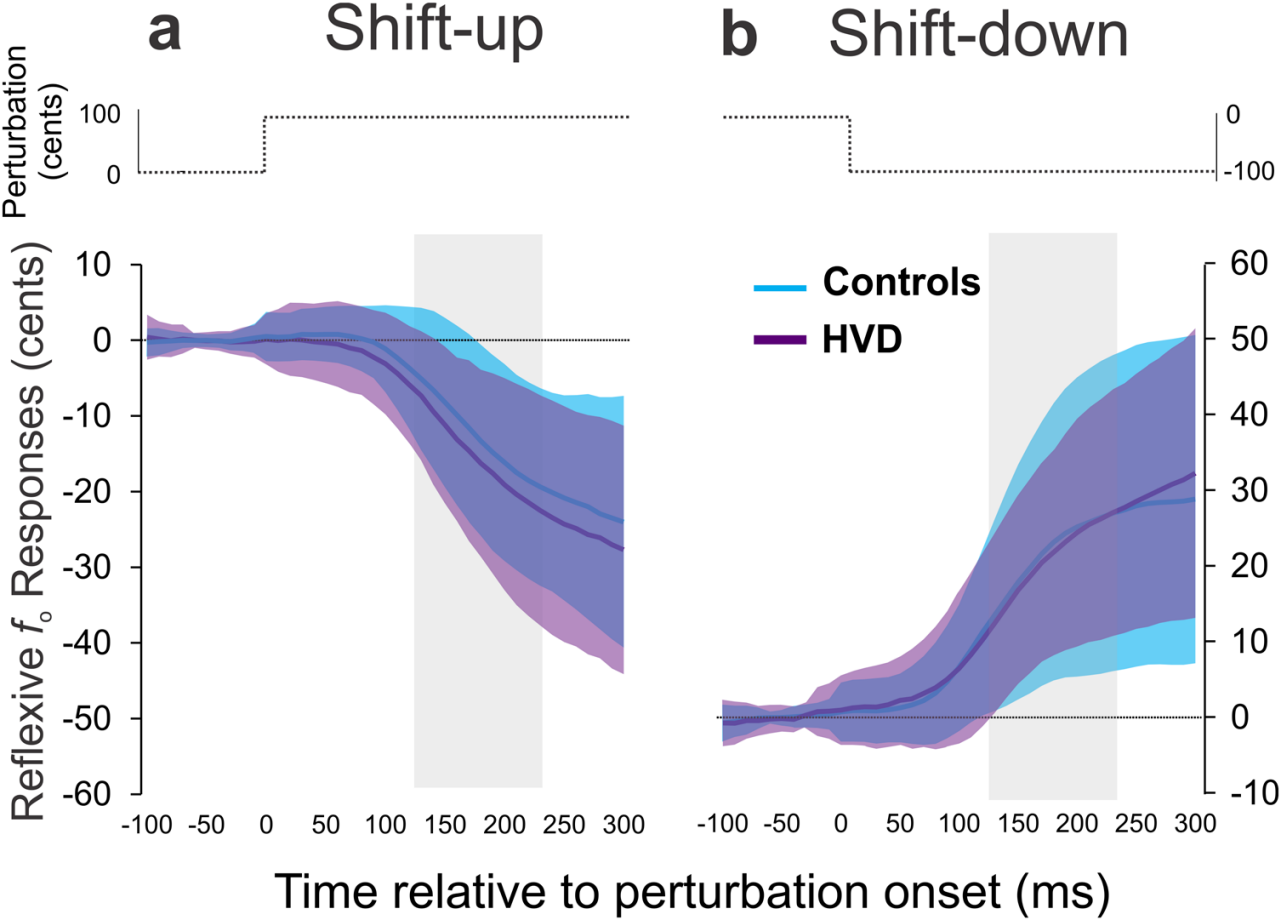
Reflexive responses. Average reflexive responses to voice fundamental frequency (*f*_o_) in cents are shown for the control group (light blue line) and hyperfunctional voice disorder (HVD) group (dark purple line) for the shift-up (panel **a**) and shift-down (panel **b**) conditions. The shaded regions in each panel indicates the group means + / − standard deviation for both the control group (light blue shading) and HVD group (dark purple shading). The grey highlighted columns show the time interval used for analysis.

A three-way mixed effects ANOVA demonstrated no main effect of group (HVD vs. controls; *df* = 1, F = 0.39, *p* = .53) or singing experience (singers vs. non-singers; *df* = 1, F = 0.00, *p* = .95) on reflexive responses, but there was a significant main effect of shift direction (shift-up vs. shift-down; *df* = 1, F = 14.28, *p* < .001, small-to-medium effect size, η_p_^2^ = 0.06). The group averages and standard deviations are plotted in Fig. 2. A post-hoc Tukey test showed that the shift-down condition resulted in a statistically larger reflexive responses (M = 21 cents, SD = 17 cents) compared to the shift-up condition (M = 14 cents, SD = 14 cents). There were small, but significant, interactions between group and shift direction (*df* = 1, F = 4.23, *p* = .04, η_p_^2^ = 0.02). The shift-down condition demonstrated statistically larger responses for both the control group (M = 21, SD = 18 cents) and HVD group (M = 21 cents, SD = 15 cents) compared to the shift-up condition for both the HVD group (M = 16 cents, SD = 14 cents) and the control group (M = 13 cents, SD = 13 cents). There were no significant interaction effects between group and singing experience (*df* = 1, F = 1.13, *p* = .29), shift direction and singing experience (*df* = 1, F = 0.74, *p* = .39), or group, shift direction, and singing experience (*df* = 1, F = 0.02, *p* = .85).

### Adaptive responses

**Figure 3:**
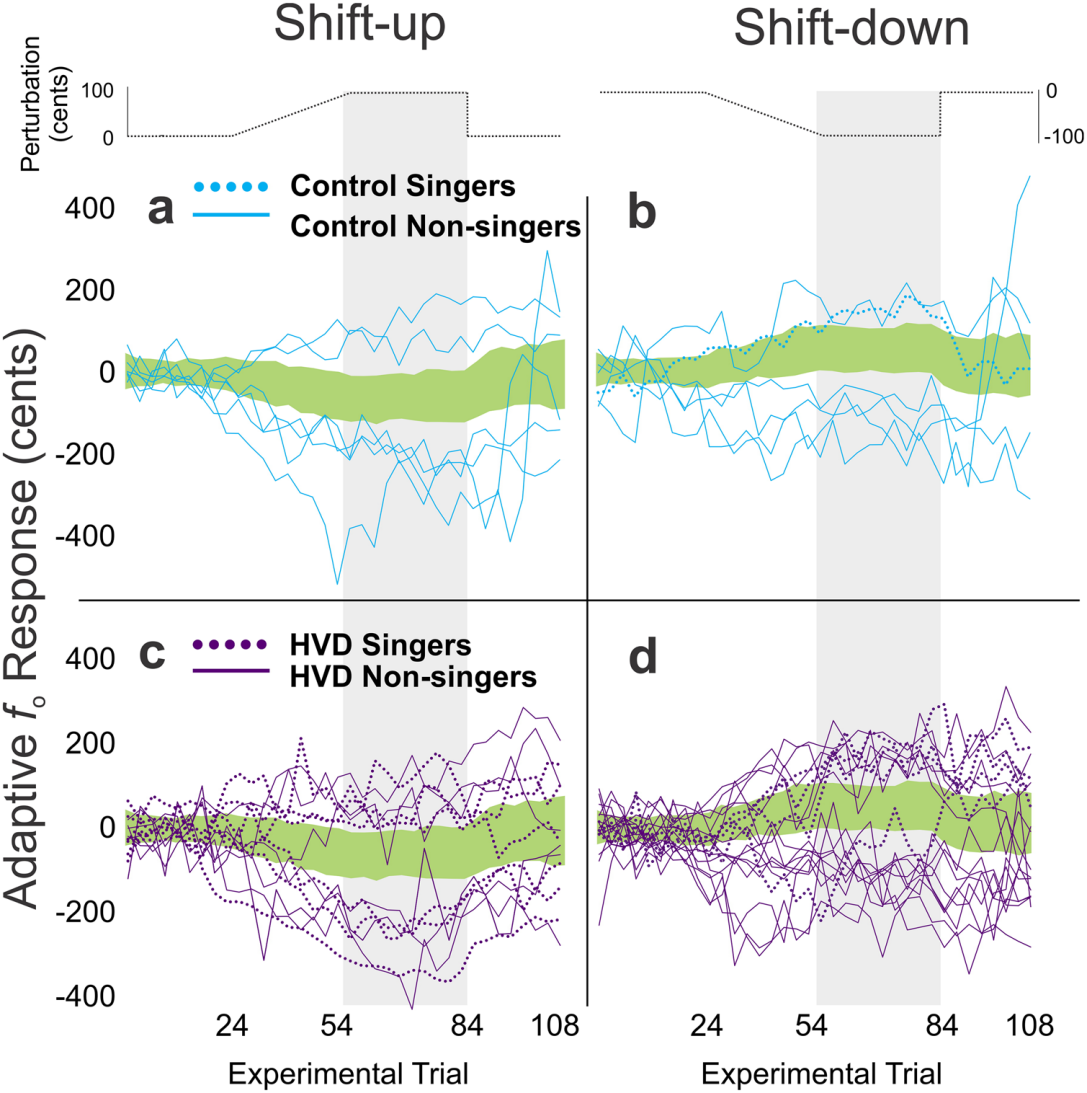
Adaptive responses. Atypical adaptive responses to voice fundamental frequency (*f*_o_) in cents are shown for the control (light blue) and HVD (dark purple) groups. The atypical responses are plotted for the shift-up (panel **a** and **c**) and shift-down (panel **b** and **d**) conditions for non-singers (solid lines) and singers (dotted lines). The green shaded region in each panel indicates the average of all responses classified as typical + / − standard deviation for both groups. The grey highlighted columns show the hold phase trials used for classification analysis.

Based on the shift-up and shift-down adaptive responses of the control group, 90th‰ z-score cutoffs were determined. These cutoffs were used to categorize all participants’ responses as typical versus atypical. By definition, the 90th‰ z-score cutoff yielded ~ 10% of atypical responses for the control group (N = 6/62 for both shift directions). In the HVD group, however, 17 – 30% of responses were classified as atypical (N = 11/62 for the shift-up condition; N = 18/62 for the shift-down condition). Atypical responses for both groups are shown in Fig. 3. A Chi-squared test for association was used to examine associations between group (HVD vs. control) and each participant’s response type, categorized as (1) an atypical shift-up response, (2) atypical shift-down responses, (3) atypical responses in both shift directions, and (4) typical responses in both shift directions. A statistically significant association was found between group and response type (*df* = 3, *X*^2^ = 14.02, N = 124, *p* = .003, large effect size, Cramer’s V = 0.33).

### Explanatory analyses

Two additional explanatory analyses were conducted using the HVD data. A linear regression model was used to examine whether auditory-perceptual ratings of the overall severity of dysphonia (see “Methods” section) were related to auditory discrimination thresholds while controlling for singing experience. Overall severity ratings were not significantly related to discrimination (*df* = 1, F = 2.08, *p* = .15), but singing experience was a significant factor (*df* = 1, F = 12.76, *p* < .001). A two-way ANOVA revealed a significant effect of singing experience (singers vs. non-singers; *df* = 1, F = 22.04, *p* < .001; large effect size, η_p_^2^ = 0.41), adaptive response type (atypical shift-up, atypical shift-down, atypical both directions, or typical both directions; *df* = 3, F = 4.82, *p* < .001; large effect size, η_p_^2^ = 0.52), and their interaction (*df* = 3, F = 12.01, *p* < .001; large effect size, η_p_^2^ = 0.40) on discrimination. A post-hoc Dunnett’s test demonstrated statistically worse discrimination for individuals with atypical responses for both shift directions (M = 112 cents, SD = 56 cents) compared to individuals with typical responses for both shift directions (M = 38 cents, SD = 20 cents), but no statistical differences between auditory discrimination thresholds for individuals with atypical shift-up responses (M = 36 cents, SD = 20 cents), atypical shift-down responses (M = 53 cents, SD = 26 cents), and typical responses.

## Discussion

The goal of the current work was to comprehensively evaluate auditory-motor control of voice fundamental frequency (*f*_o_) in a large group of singers and non-singers with HVDs and a well-matched control group. Based on early work suggesting that individuals with HVDs may have disruptions in auditory-motor control, we expected that both singers and non-singers in the HVD group would demonstrate: (1) worse discrimination of voice; (2) greater reflexive response magnitudes; and (3) greater occurrences of atypical adaptive responses compared to controls. The experimental results confirmed the first and third hypotheses, but there was no observed effect of group on reflexive response magnitudes.

The findings for auditory discrimination support the first study hypothesis: both singers and non-singers with HVDs demonstrated worse discrimination than controls. Together with previous findings, this strengthens evidence that individuals with HVDs have difficulty in detecting changes in auditory feedback, regardless of whether sounds are externally-generated (i.e., pure tones^23^) or internally-generated (i.e., their own voice). Although the use of voice stimuli allowed for the investigation of auditory input directly related to each speaker’s auditory-motor *f*_o_ control, this methodological choice may have introduced variability due to degraded vocal quality in the HVD group. However, the results of our explanatory analysis demonstrated no association between overall severity of dysphonia and discrimination thresholds, suggesting that voice quality is unlikely to have driven the poorer discrimination in the HVD group.

Conflicting with evidence from a prior study^24^ that informed the second study hypothesis, no statistical differences were found for reflexive *f*_o_ response magnitudes between the HVD and control groups (regardless of singing experience). This result is likely driven by the substantial differences in methodology between the two studies. The present study aimed to examine a large group of speakers with HVDs (N = 62) and a hearing-, sex-, and age-matched control group (N = 62), whereas the previous study examined reflexive responses from only 10 speakers with HVDs (aged 21–64 years) and 17 controls (aged 20–30 years). The prior work reported greater reflexive *f*_o_ response magnitudes in the HVD group^24^, but these may have resulted from the small sample size or age characteristics of the HVD group (reflexive response magnitudes increase with aging^36,37^). The reflexive response methods may also explain the disparate results between the studies. The current study employed a sudden, sustained perturbation^31,38,39^ and used a specific 120–240 ms window after shift onset to best capture the feedback system response^31,39,40^. The earlier study, which examined a 0–500 ms window following a brief voice shift, may have included components of a second, voluntary vocal response that is thought occur after ~ 265 ms^41^. Further, a very large shift was applied in the previous work (700 cents^24^) when compared to prior reflexive *f*_o_ paradigms (ranging from 50 to 200 cents^29–31^). The size of the voice shift is known to influence response magnitudes, presumably because speakers may no longer perceive a drastically altered voice as their own^29^. Although no differences were found as a function of group in the current study, statistically larger responses were seen for the shift-down compared to the shift-up condition. This finding is in agreement with prior work in typical speakers^42^. Overall, our work provides robust evidence that reflexive *f*_o_ responses are intact in individuals with HVDs and that the auditory-motor feedback system can generate typical corrective motor plans for 100 cents shift magnitudes. Importantly, we found that the average discrimination threshold (error detection) was below 100 cents in both groups, so future studies should examine whether corrective motor plans remain intact in HVDs for smaller shift magnitudes.

In line with a preliminary study^25^ and the third study hypothesis, adaptive *f*_o_ responses revealed greater numbers of atypical responses in the HVD group compared to controls. Atypical responses were found for both singers and non-singers. As seen in the prior study, responses in the HVD group included ‘following’ responses (in the direction of the perturbation) and very large compensatory responses (opposing the perturbation) so group averages were unable to capture the atypical responses (S2). Instead, a z-score method was used to establish a 90th‰ cutoff, which resulted in an expected 10% of atypical responses in the control group but up to three times as many atypical responses in the HVD group. Since HVDs are heterogeneous^3,9^, we anticipated a range in adaptive responses in the HVD group (both typical and atypical); however, the statistically greater frequency of atypical responses in the HVD group implicates disruptions in auditory-motor integration as a contributor to HVDs, perhaps indicative of an auditory-motor phenotype^21^. Explanatory analyses revealed that poorer auditory discrimination was associated with atypical adaptive responses in the HVD group with a large effect size. For instance, the participants with atypical responses in both directions had an average discrimination threshold of 1.12 cents, which was larger than the magnitude of the experimental voice shift that was implemented. Future work is necessary to determine whether atypical adaptive responses in individuals with HVDs are related to the relationship between their average discrimination threshold and the magnitude of the experimental shift. Although a causal relationship cannot be determined from the present study, the results suggest that the atypical auditory-motor adaptive responses and the motor production symptoms inherent in HVDs may have an underlying basis in auditory function. This motivates comprehensive assessment of peripheral and central auditory function in individuals with HVDs.

The current work included both singers and non-singers, expanding upon prior work that did not consider singing experience (which has been shown to impact auditory-motor control in all three tasks examined^32,34,43^). Since the HVD and control groups were matched for singing experience, singing experience was included as a variable in all analyses. As expected, singers demonstrated a greater number of typical adaptive responses and better auditory discrimination compared to non-singers, but no impact of singing experience was found on reflexive responses in either speaker group. Prior work has reported reduced reflexive responses in singers as well as no differences in response magnitudes between singers and non-singers^35,43^. The former study was conducted in speakers of a tonal language, which could play a role in differing findings, while the latter and current work were conducted in non-tonal language speakers.

This study investigated a comprehensive set of tasks related to auditory-motor control in HVDs and a well-matched control group, but there are limitations. The aim of this investigation was to characterize the auditory components of sensorimotor control of voice *f*_o_; yet typical speakers show sensory preferences (i.e., auditory or somatosensory) during sensorimotor integration of speech^44^, so disruptions to auditory-motor control in HVDs may interact with speaker-specific sensory weighting. Examining somatosensory motor control in HVDs would further inform the inter-speaker variability in auditory-motor integration observed here. The current work also did not include laryngeal imaging. Incorporating laryngeal imaging in subsequent investigations would elucidate relationships between laryngeal-motor features and auditory function to determine if auditory-motor impairments are associated with specific subtypes of HVDs. Another factor to consider is the inclusion of several perturbation tasks within one study session, which could lead to habituation to auditory perturbations. However, the experiment was designed such that the adaptive responses (consisting of gradual perturbations) were all completed first and the reflexive responses (with clearly perceivable voice *f*_o_ perturbations) were completed last, so we do not expect the session duration to have confounded these results. Lastly, although the speaker groups were carefully matched for several variables, there were slight differences in age between the two speaker groups. This difference was not statistically significant for singers, but the non-singers with HVDs had statistically larger age (M = 40.5 years) than non-singers without HVDs (M = 32.8 years; *df* = 63, T-value = 2.26, *p* = .03). For auditory discrimination and adaptive responses, group differences were found regardless of singing experience so it is unlikely that slight age differences in the non-singer group influenced our findings. If age had impacted our reflexive response results for the non-singers, larger reflexive responses may have been observed for non-singers with HVDs since reflexive response magnitudes increase with aging^36,37^; yet we saw no differences in reflexive responses between groups for both singers and non-singers.

In sum, the current study provides strong evidence for auditory-motor disruptions in a substantial portion of both singers and non-singers with HVDs. The HVD group demonstrated worse auditory discrimination and a greater frequency of atypical adaptive responses, which suggests impairments in how auditory feedback errors are detected and how the auditory-motor feedforward plan is updated in HVDs. These findings represent a shift in our understanding of HVDs by highlighting the involvement of audition in hyperfunctional voice symptoms and laying the groundwork for future treatment studies for HVDs.

## Methods

All experimental protocols were approved by the Boston University and University of Washington Institutional Review Boards. All study participants provided informed consent prior to the study, and all experiments were performed in accordance with relevant guidelines and regulations from the Boston University or the University of Washington Institutional Review Board.

### Participants

A total of 124 individuals with and without hyperfunctional voice disorders (HVDs) participated in an observational cohort study (see Supplementary file S1). The HVD group consisted of 33 cisgender non-singers (26 females, 7 males) and 29 cisgender singers (28 female, 1 male). The control group consisted of 33 cisgender non-singers, 28 cisgender singers, and one genderqueer singer who were all sex-matched to the HVD group. The HVD group ranged from 18 to 67 years old (average age of 33.6 years, standard deviation of 14.0 years) and the control group ranged from 18 to 69 years old (average age of 28.4 years, standard deviation of 11.2 years). Participants were classified as singers if they had 5 or more years of formal training in vocal performance.

All individuals with HVDs were diagnosed by a laryngologist based on a comprehensive voice evaluation including videolaryngoscopy at either the Boston Medical Center, the Massachusetts General Hospital Voice Center, or the University of Washington Medical Center. None of the recruited individuals with HVDs had a history of neurological disorders or other speech, language, and hearing disorders. All control participants reported no history of neurological, voice, speech, language, or hearing disorders. No participants were receiving hormone therapy or other medications that impact the voice.

Due to the impact of hearing on auditory processing^45^, all participants underwent hearing threshold testing with insert earphones or headphones on a GSI 17 or GSI 18 audiometer (Grason-Stadler, Littleton, MA). Participants were included if they passed the hearing screening at thresholds appropriate for their age. For individuals who were under the age of 50, the passing threshold was 20 dB HL at 250 Hz, 500 Hz, 1000 Hz, 2000 Hz, and 4000 Hz^46^. For individuals who were aged 50 or older, the passing threshold was 25 dB HL at frequencies of 1000 Hz and below and 40 dB HL at frequencies above 1000 Hz^47^. Five of the participants with HVDs had a threshold within a 10 dB range of the cutoff criteria and were included in the study with five hearing-matched control participants. No participants wore hearing aids.

A priori power analyses demonstrated that 60 speakers with HVDs and 60 controls would allow detection of small-to-medium effect sizes (e.g., η_p_^2^ = 0.1) with α = 0.05 and power of 80%.

### Data collection

Data were collected in a sound-attenuated booth across two study sessions lasting 2–3 h each at either Boston University or the University of Washington. The experimental hardware, software, setup, and study protocol were identical between the two study locations. An omni-directional ear-set microphone (Shure MX153) at approximately 45 degrees from the midline and 7 cm away from the corner of the mouth was used to record all speech signals. The microphone gain was adjusted with a preamplifier (RME Quadmic II) and was digitized with a soundcard (MOTU Ultralite-mk3 Hybrid or RME Fireface UCX). For the majority of the participants (N = 120), an Eventide Eclipse V4 Harmonizer was used to create experimental shifts in voice fundamental frequency (*f*_o_) with a processing delay between 10 and 30 ms^39^. For four participants, Audapter software^48^ was used to create shifts in voice *f*_o_ with a processing delay up to 45 ms^31^. The processed signal was amplified with an earphone amplifier (Behringer Xenyx Q802) and auditory feedback was administered via Etymotic ER-2 insert earphones or Sennheiser HD 280 Pro headphones.

Prior to all participant sessions, the software and hardware systems were calibrated using a 2 cc coupler (Type 4946, Bruel and Kjaer Inc) connected to a sound level meter (Type 2250A with a Type 4947 ½” Pressure Field Microphone, Bruel and Kjaer). For the reflexive and adaptive responses, the earphone intensity was calibrated such that a 1 kHz tone played from a handheld recorder (Olympus LS-10 Linear PCM Recorder) positioned 7 cm from the microphone yielded an amplification of + 5 dB relative to the microphone signal. For the auditory discrimination task, the same equipment was used to calibrate earphone output to be 75 dB SPL, regardless of the intensity of the speaker’s voice recording.

All speakers completed the experimental tasks in the following order: adaptive responses, auditory discrimination, a reading task, and reflexive responses. For the adaptive and reflexive responses, the shift order (shift-up or shift-down) was randomized across participants. The adaptive responses were collected first since they involve gradual changes in voice *f*_o_, whereas the reflexive responses were deliberately collected last due to the clear presence of shifts in voice *f*_o_.

#### Auditory discrimination

**Figure 4:**
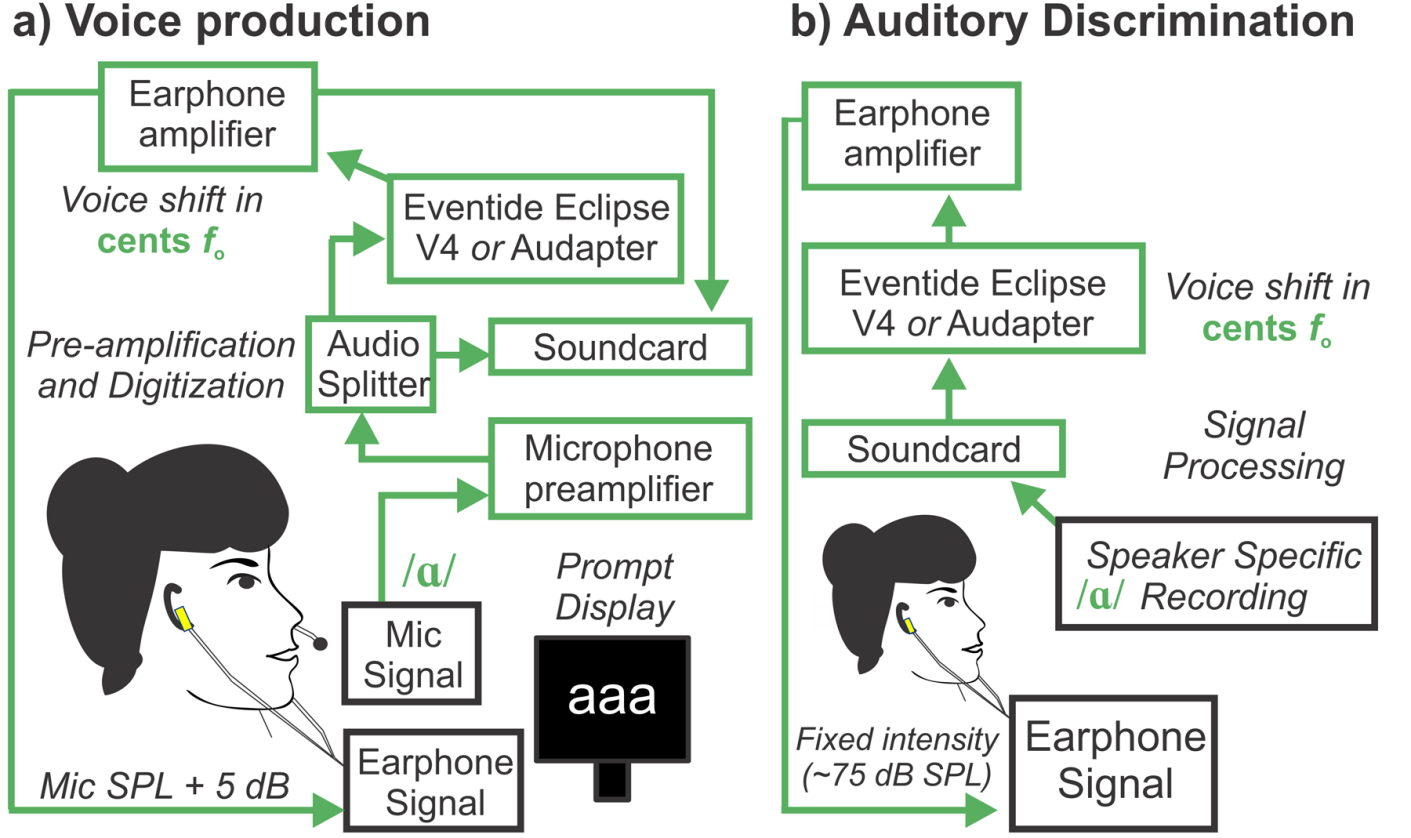
Experimental setup. Hardware and software setup are shown for the voice production (panel **a**) and auditory discrimination (panel **b**) tasks involving shifts in auditory feedback of fundamental frequency (*f*_o_) in cents (100 cents = 1 semitone). Voice production tasks consisted of speakers actively producing an/ɑ/vowel, with prompts on a computer display, during *f*_o_ perturbations in auditory feedback administered with a 5 dB increase in sound pressure level (SPL) relative to the microphone (mic) signal. The auditory discrimination task consisted of making judgments by listening to shifts in voice *f*_o_ played back at a set level of 75 dB SPL.

Auditory feedback for the auditory discrimination task was administered at a set level of 75 dB SPL (panel b; Fig. 4). Discrimination thresholds were quantified with a just-noticeable-difference experiment with a one-up, two-down staircase procedure that used a 1:1 up-down ratio to obtain a target threshold of 70.71%^49^; this experiment lasted 4.3 min on average (standard deviation = 0.9 min).

Prior to the experiment, participants were asked to produce a steady /ɑ/ vowel for 2–3 s (s) and their voices were recorded with Praat software^50^. A steady 500-ms portion of each produced vowel was extracted. These were used as speaker-specific stimuli for the listening task. During the listening task, participants were presented with pairs of their /ɑ/ recordings and asked to judge whether the two stimuli sounded the ‘same’ or ‘different’ in terms of their pitch. Each trial consisted of one stimulus that was a reference (the original recording) and one stimulus with a shift in *f*_o_, which was applied based on the staircase procedure logic (presented in randomized order). For all participants, the initial *f*_o_ change applied to the shifted stimulus was + 50 cents (100 cents is equivalent to one semitone), with a 4 cent change in direction following two correct responses (decreasing) or one incorrect response (increasing). In 20% of trials (‘catch trials’), the reference stimulus was played twice to ensure attention to the task. Catch trial responses were not used in the staircase logic, but all participants had above chance catch trial accuracy (> 50%). The full experiment included either ten reversals (i.e., changes in the direction) or 60 trials, whichever occurred first.

#### Reflexive and adaptive responses

During the tasks to elicit reflexive and adaptive *f*_o_ responses, participants were actively voicing while their auditory feedback was experimentally manipulated (panel a; Fig. 4). Auditory feedback was administered with a 5 dB increase in sound pressure level (SPL) relative to the microphone signal^39^. For both tasks, participants were instructed to sustain a steady /ɑ/ vowel for 2–3 s for 108 trials per condition. Shifts in voice *f*_o_ were applied in cents. The inter-trial interval was randomly jittered between 1 and 3 s to prevent rhythmic cues, and each task condition lasted 10 min.

The reflexive response task consisted of two conditions: shift-up and shift-down. Each condition had 84 trials with typical feedback and 24 trials with a sudden onset voice shift of either + 100 cents (shift-up) or – 100 cents (shift down) in voice *f*_o_. In order to limit habituations to the shifted feedback, there were always at least three typical feedback trials between each shifted trial. In the reflexive response tasks, voice shifts did not begin at the start of the trial. During shifted trials, voice shifts occurred randomly between 0.5 and 1 s after voicing onset to allow the voice to stabilize before the unexpected feedback shift, and remained for the duration of the trial as in prior work^31,38,39^. The two conditions were completed in counterbalanced order across participants.

The adaptive response task consisted of three conditions: shift-up, shift-down, and control. The conditions with voice shifts (shift-up and shift-down) were completed first or third, in counterbalanced order across participants. The control condition was always completed second. The voice shift conditions involved four ordered phases: ‘baseline’: 24 trials of unaltered feedback; ‘ramp’: 30 trials with gradual increases (shift-up) or decreases (shift-down) of 3.3 cents in the *f*_o_ of the auditory feedback each trial; ‘hold’: 30 trials with the voice shifts maintained at + 100 cents (shift-up) or – 100 cents (shift-down); and ‘after-effect’: 24 trials of unaltered feedback. All voice shifts were applied at the beginning of each trial and were maintained for the full period of vocalization.

#### Reading task

All but one participant completed a reading task that included The Rainbow Passage^51^. Audio recordings were recorded with SONAR Artist (Cakewalk, Inc.) software.

### Data analysis

For auditory discrimination, the threshold was estimated by calculating the average *f*_o_ shift values in cents across the last six reversals for each participant^27^.

For voice production tasks, an *f*_o_ trace was extracted for each trial via an autocorrelation method using Praat^52^ and MATLAB^53^ scripts. For the reflexive responses, the 120–240 ms portion of each shifted trial was extracted (with 0 ms corresponding to the onset of the voice shift) as done in prior work^31,39^. This region was selected to reflect the feedback control system response^40^. The raw values in Hz were converted to cents using the average across the 100 ms immediately preceding the voice shift as a baseline for each trial. Voice *f*_o_ in cents during the 120–240 ms portion was averaged across all shifted trials into a single trace for each speaker for each condition (shift-up and shift-down). The average across the *f*_o_ values in the 120–240 ms trace (sampled every 10 ms) was termed as the ‘reflexive response’ and was used for analyses for each condition. The additive inverse was applied to the shift-up average to compare magnitudes across shifted conditions.

For the adaptive responses, the 40–120 ms portion of each trial was extracted (with 0 ms corresponding to the start of vocalization) and the average voice *f*_o_ was calculated in Hz. This early portion of vocalization was used to capture the feedforward control system response^40^. The average *f*_o_ values across the baseline trials in each condition were used as reference values to convert the average *f*_o_ of each trial to cents for each associated condition (shift-up, shift-down, and control). To account for natural variability in voice *f*_o_, the control condition was then subtracted from the two voice shift conditions^54^. The resulting average values for the ‘hold’ phase trials were termed ‘adaptive responses’ and used to examine the degree of adaptation to the voice shifts for the shift-up and shift-down conditions^54^. When examining group-level averages for hold phase trials, the groups showed comparable responses; however, individual traces revealed large individual variability in the HVD group compared to the control group for both shift conditions. This included ‘following’ responses (in the direction of the perturbation) and very large ‘compensatory’ responses (opposing the perturbation, with magnitudes much larger than seen in speakers with typical voices), which had been observed in preliminary work as well^25^. For this reason, comparing group averages could not capture the nature of auditory-motor disruptions in the adaptive response (see S2).

To capture these differences in adaptive responses, z-scores were calculated and used to classify individual responses as either ‘typical’ or ‘atypical’ for the HVD and control groups. Z-scores were computed by calculating the mean adaptive responses for the full control group (singers and non-singers) and setting a 90th‰ cutoff value for classification for the shift-up and shift-down conditions. Using the z-score cutoff values for the shift-up (z = 1.46) and shift-down (z = 1.52) conditions, all participant responses were classified as either ‘typical’ (below the 90th‰ cutoff) or ‘atypical’ (greater than the 90th‰ cutoff). This classification yielded a total count for typical and atypical response types for each group.

A blinded voice-specializing speech-language pathologist provided ratings of overall severity of dysphonia using the Consensus Auditory-Perceptual Evaluation of Voice (CAPE-V^55^) for the HVD group based on a recording of the Rainbow Passage^51^ (see Supplementary file S1). Of the 62 individuals with HVDs, one participant did not have a Rainbow Passage recording, and their speech was evaluated based on sustained vowel recordings from the baseline phase of the experimental task. The overall severity ratings (0 = no dysphonia, 100 = maximum severity of dysphonia) ranged from 0 to 55.1, with an average of 13.5 and standard deviation of 12.0. Approximately ~ 15% of samples (N = 9/62) were repeated for reliability and a Pearson’s correlation coefficient showed a strong association between ratings (r = 0.86).

### Statistical analysis

All statistical analyses were performed using Minitab 19 software^56^. Alpha levels of *p* < .05 were set a priori and used to determine statistical significance.

For the discrimination and reflexive response tasks, analyses of variance (ANOVAs) were used to examine results. For discrimination analyses, a two-way ANOVA was used to assess the effect of group (HVD vs. control) and singing experience (non-singer vs. singer) as fixed factors, and their interaction, on auditory discrimination. A three-way mixed effects ANOVA was used to examine reflexive response magnitude with fixed factors of group (HVD vs. control), singing experience (non-singer or singer), shift direction (shift-up vs. shift-down), and their interactions, with participant as a random factor. Factor effect sizes were quantified using the squared partial curvilinear correlation η_p_^2^ and post-hoc Tukey tests were used to determine the direction of relationships.

For the adaptive responses, a Chi-squared test for association was used to assess the counts of atypical shift-up responses, atypical shift-down responses, atypical responses in both directions, and typical responses in both directions by group (HVD vs. control). Effect size was quantified using Cramer’s V.

In addition to the planned analyses, two additional explanatory analyses were performed on data from the HVD group to examine: (1) if auditory discrimination was associated with overall severity of dysphonia of the voice; and (2) if auditory discrimination was associated with adaptive response type. For the first analysis, a linear regression model was fit to auditory discrimination thresholds, with overall severity of dysphonia (via CAPE-V) and singing experience (non-singer vs. singer) as explanatory variables. For the second analysis, a two-way ANOVA was used to assess the effect of singing experience, adaptive response type (atypical shift-up, atypical shift-down, atypical both directions, or typical), and their interaction on discrimination thresholds. A post-hoc Dunnett’s test was used to determine the direction of relationships by comparing the discrimination thresholds for atypical shift-up, atypical shift-down, and atypical both directions to the typical response type (control).

## Supplementary Information

Supplementary Information S1.

Supplementary Information S2.

Supplementary Information Legend.

## Data Availability

All data needed to evaluate the conclusions in the paper are present in this published article (and its Supplementary Information files).
