# Supplementary material for: Impaired auditory discrimination and auditory-motor integration in hyperfunctional voice disorders
Source: Sci Rep. 2021 Jun 23;11:13123. doi: 10.1038/s41598-021-92250-8 (PMC8222324; doi:10.1038/s41598-021-92250-8)
Supplement: Supplementary file 3 — Supplementary Information Legend. [file 41598_2021_92250_MOESM3_ESM.docx]

**Supplementary Information**

**S1. Group characteristics and data**. Control group characteristics (age, sex, gender, and singing experience), hyperfunction voice disorder (HVD) group characteristics (age, sex, gender, singing experience, medical diagnosis, and Consensus Auditory-Perceptual Evaluation of Voice: CAPE-V overall severity ratings), auditory discrimination data (discrimination thresholds), reflexive response data (mean shift-up response and mean shift-down response), and adaptive response data (mean shift-up response, mean shift-down response, and response type).

**S2. Group-level adaptive responses.** Group-level average adaptive responses to voice fundamental frequency (*f*_o_) in cents are shown for the control group (light blue line) and HVD group (dark purple line) for the shift-up perturbation (panel a) and the shift-down perturbation (panel b). The shaded region in indicates the average of all +/- standard deviation for both the control group (light blue shading) and HVD group (dark purple shading).
